# *Schizosaccharomyces japonicus* Yeast Poised to Become a Favorite Experimental Organism for Eukaryotic Research

**DOI:** 10.1534/g3.113.007187

**Published:** 2013-10-01

**Authors:** Amar J. S. Klar

**Affiliations:** Gene Regulation and Chromosome Biology Laboratory, National Cancer Institute, Center for Cancer Research, National Institutes of Health, Frederick, Maryland 21702-1201

**Keywords:** *Schizosaccharomyces japonicus*, fission yeast, fast growing, rapid meiotic analysis, organism conducive for research

## Abstract

Both budding yeast *Saccharomyces cerevisiae* and fission yeast *Schizosaccahromyces pombe* have been very popular organisms used for biological research with eukaryotes for many decades. Judging from the fission yeast *Schizosaccharomyces japonicus* DNA sequence determined 2 years ago, this species is evolutionarily very much unrelated to the commonly used yeasts for research. Indicating evolutionary divergence, the *S. japonicus* makes 8-spored asci and mitosis occurs with a partial breakdown of nuclear membrane whereas the other yeasts make 4-spored asci and cells divide without nuclear breakdown. The commonly used yeast species exhibit a generation time between 1.5 and 2.0 hr, and their genetic cross takes a period of more than 7 working d. As described here, a generation time of only 63 min and meiotic analysis completed in just 2.5 d, the *S. japonicus* fission yeast is predicted to become a choice organism for future research on the biology of eukaryotes.

In the 1970 edition of the *Molecular Biology of the Gene*, Dr. James D. Watson (Watson 1970) remarked, “even if our primary interest is the human cell, this may be the time for many biologists to work with organisms like yeast.” Examples abound whereby basic research in biology has been fueled by studies introducing new organisms and/or new techniques for research. Such developments have permitted the investigators to ponder new questions not possible to address by the research of previously exploited organisms and techniques. The popularization of the roundworm research to study the development of multicellular organisms by Brenner (2003) and the development of genetic engineering by a DNA-mediated transformation procedure of *Saccharomyces (Sc.) cerevisiae* by Fink and others (Hicks *et al.* 1979; Hinnen *et al.* 1978) are just two such prominent examples that have sped the progress of biological research in the last four decades. Here I describe extremely advantageous features that are bound to help popularize biological research with *Schizosaccharomyces* (*S*.) *japonicus*, the fission yeast whose genome sequence was determined two years ago (Rhind *et al.* 2011).

The budding yeast *Sc. cerevisiae* and fission yeast *Schizosaccahromyces* (*S*.) *pombe* are inexpensive model eukaryotic organisms used for biological research. Notably, most research areas of yeast could not be investigated in a timely manner in higher eucaryotes. Because mechanisms of many biological pathways are evolutionarily conserved in eukaryotes, prominent among them being the cell cycle (Hartwell 2002; Nurse 2002), these two organisms have led the way to defining new principles of eukaryotic biology. The knowledge gained with model organisms has been subsequently applied to fuel investigations of higher organisms, such as mammals. We recently published a study on *S. japonicus* variety *versatilis* in which the process of asymmetric cell division through the DNA strand-specific imprinting/segregation mechanism was described (Yu *et al.* 2013). During the study with this highly diverged yeast, compared with his experience working with the *S. pombe* and *Sc. cerevisiae* yeasts, this author noted important features highly conducive for conducting research with *S. japonicus*. These features, which are not presently appreciated by yeast researchers at large, are described herein. Because this species is not well represented in the literature, presently its potential has not been recognized.

## Materials and Methods

### Strains and culture conditions

The genotype of strains used is listed in Table 1. Yeast extract agar (YEA) medium was used for cultures growth; Pombe minimal adenine (PMA) medium was used to induce meiosis and sporulation; octad analyses were carried out according to the previously described procedures used for research with *S. pombe* cultures (Moreno *et al.* 1991).

**Table 1:** *S. japonicus* strains genotype

Strain	Iod	Genotype	Source
Sjk2	+	*mat1-P*, *mat2/3Δ*	Yu *et al.* (2013)
Sjk3	+	*mat1-M*, *mat2/3Δ*	Yu *et al.* (2013)
Sjk10	+	*mat1-P*, *mat2/3Δ*, *ade6-dam-E*, *ura4-D3*	This study
Sjk17	+	*h^+^*, *mat-2017*, *mrc1Δ*::*Nat*	This study
Sjk19	+	*mat1-M*, *mat2/3Δ*, *mrc1Δ*::*Nat*	This study
NIG6701	-	*h^+^*, *mat-2017*, *mrc1Δ*::*Nat*	Furuya and Niki (2012)

Iod indicates iodine vapor−staining colony phenotype when cells of opposite mating type are mated for meiosis and sporulation. The symbol *Δ* indicated deletion of the gene. The *mat2*/*3Δ* donor loci−deleted strains were derived from Sjk4, which is homothallic for the mating-type switching phenomenon (Yu *et al.* 2013). *Nat* is a Nourseothricin gene cassette that replaced the *mrc1* gene in the genome; it confers the Nourseothricin antibiotic resistance to yeast. The *ade6*-*damE* and *ura4*-*D3* markers were derived from a stock provided by Dr. H. Niki.

### Cytology

Cells pictures were taken with ZeissAxioobserver Z1 with LD plan nesofluar microscope.

## Results and Discussion

### The *S. japonicus* var. *versatilis* genetic cross completed in 60 hr

**Figure 1:**
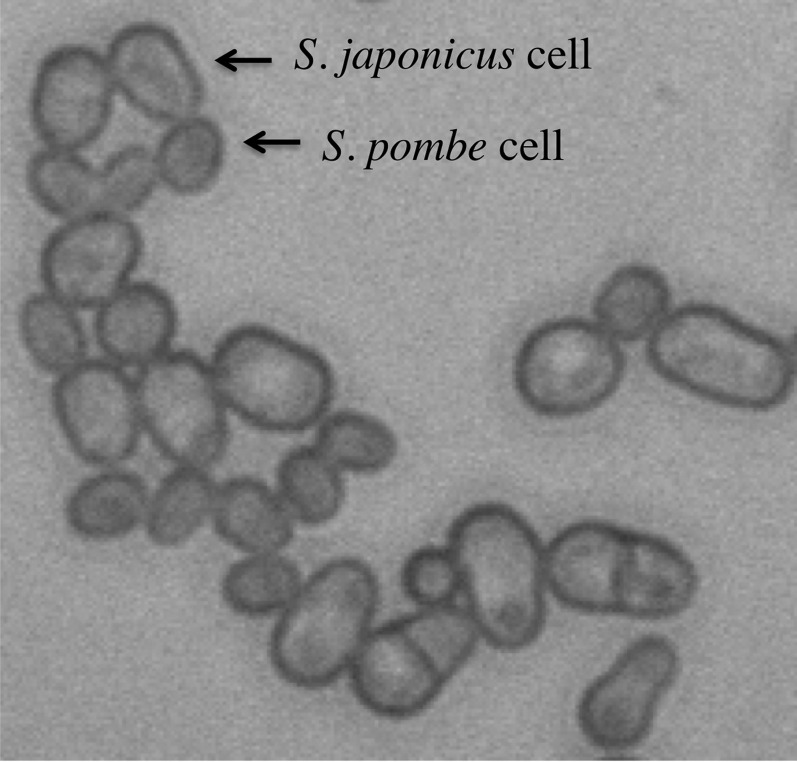
Size differences of *S. japonicus* var. *versatilis* and *S. pombe* cells. The larger cells of *S. japonicus* and the smaller ones of *S. pombe* are indicated.

The *S. japonicus* organism consists of haploid cells, genome comprising of three chromosomes, and DNA content of 11.5 Mb (Rhind *et al.* 2011); these features are similar to those of *S. pombe* species. The cell size of *S. japonicus* is several folds bigger than that of *S. pombe* (Figure 1).

**Figure 2:**
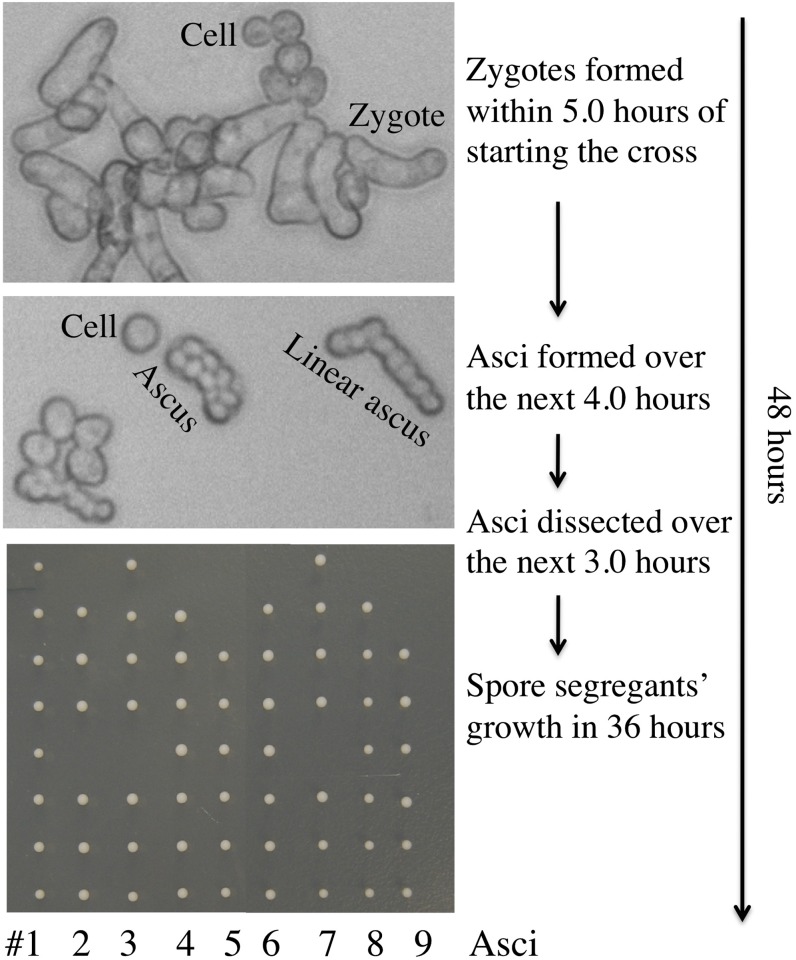
A timeline of the formation of spore segregants by the Sjk2 and Sjk3 heterothallic strains cross. The colonies of segregants of each one of nine asci, arranged in a vertical row, are displayed in the bottom panel.

Like *S. pombe* (Klar 2007), *S. japonicus* cells alternate between two cell types, called P (plus)and M (minus), which are controlled by the *mat1-P* and *mat1-M* alleles, respectively, of the mating-type (*mat1*) locus (Furuya and Niki 2009; Rhind *et al.* 2011; Yu *et al.* 2013). When cells of opposite mating types, strains Sjk2 (*mat1-P*, *mat2*/3*Δ*, *iod^+^*; see Table 1 for a description of the complete genotype) and Sjk3 (*mat1-M*, *mat2*/3*Δ*, *iod^+^*), were mixed on the mating/sporulation medium used for the *S. pombe* research, *S. japonicus* var. *versatilis* cells formed zygotes by mating within 5 hr and produced matured 8-spored asci over the next 4 hr at the 30° incubation temperature we used to induce mating, meiosis and sporulation (Figure 2). The asci were dissected by micromanipulation in the next 3 hr. After dissection, the spore segregants grew into colonies in only 36 hr with the size suitable for genotype determination. Thereafter, we determined the genotype of the segregants in the next 12 hr, including their mating type, by replica plating to nutritional dropout medium (to be described in the section *The japonicas and versatilis varieties are genetically compatible*). Thus, meiotic analysis was completed in a mere 60-hr period from the time the cross was started (Figure 2). Because sporulation of *versatilis* occurs at 30° in 9 hr and the asci start to fall apart in the following couple of hours, for logistical reasons we slowed the process by sporulating cultures at 25°. This way, a genetic cross started late in the afternoon was found to be perfectly poised for ascus dissection as first thing the following morning.

### The japonicus and versatilis varieties are genetically compatible

An extremely useful, powerful and simple procedure employed in research with *S. pombe* consists of staining sporulated colonies by exposing them to iodine vapors for two minutes (Bresch *et al.* 1968). The ascospores synthesize the starch-like compound, but the mitotically multiplying cells do not. As a consequence, sporulating colonies stain black with this procedure but the vegetatively growing colonies remain unstained. Likewise, the sporulated colonies of the *S. japonicus* var. *versatilis* stock that we employed for our cell differentiation study stained with iodine vapors (Yu *et al.* 2013). By exploiting this procedure, we recently described isolation of switching-incapable (designated “heterothallic”) mutants of *S. japonicus* var. *versatilis*. These were isolated simply by screening for non-staining phenotype of spontaneously generated mutant derivatives with the iodine-exposure procedure. These mutants, strains Sjk2 and Sjk3, resulted from spontaneous deletion of both donor *mat2* and *mat3* loci from Sjk4 (homothallic), which are used as the source of genetic information for switching *mat1* through recombination (Yu *et al.* 2013). A handful of recent publications have described research with another variety, named *S. japonicus* var. *japonicus*. Notably, the *japonicus* variety sporulated colonies do not stain with the iodine procedure (Furuya and Niki 2009). Lacking the ability to stain, isolation of heterothallic mutants from *S. japonicus* var. *japonicus* stock necessitated the determination of the mating type of approximately 20,000 mutagenized colonies, assaying them individually by microscopic analysis (Furuya and Niki 2009). This highly useful staining procedure is also not applicable for research with the *Sc. cerevisiae* yeast.

**Figure 3:**
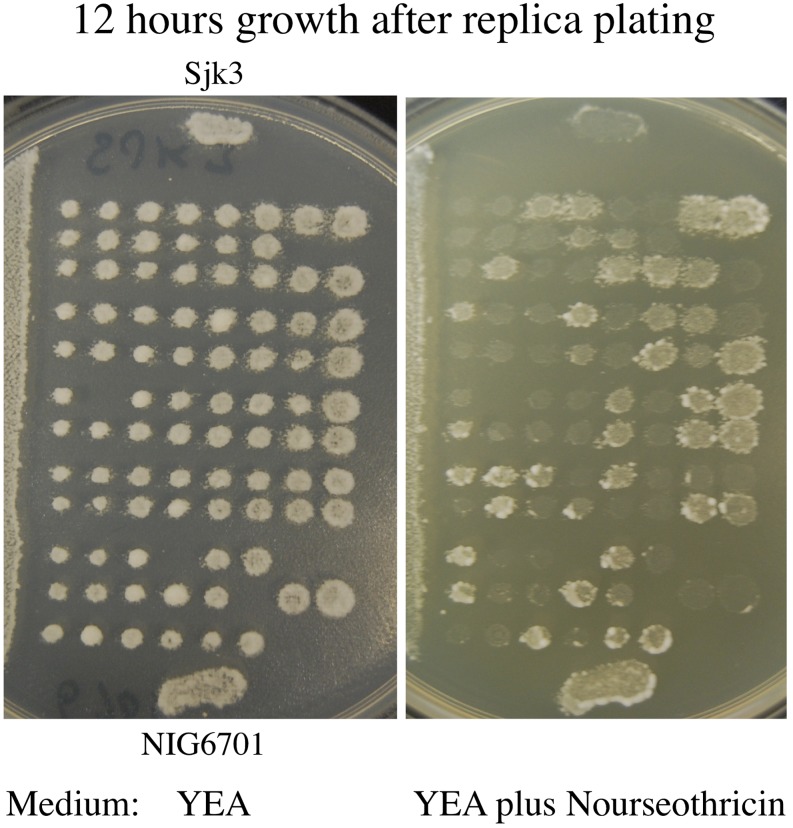
Octad analysis of heterothallic strains of Sjk3 (*mat1-M*, *mat2*/3*Δ*; variety *versatilis*) crossed with NIG6701 (*h^+^*, *mrc1Δ*::*Nat*; variety *japonicus*). Patches of cells from both parental stocks were spotted on agar surface to compare their phenotype with those of segregants of their cross. Here, segregants from individual asci were spotted in horizontal rows. The picture shows 12-hr growth after replica plating on the indicated medium.

We crossed heterothallic strain Sjk3, our strain of the *versatilis* variety, with the NIG6701 (*mat1-P*, *mat-2701*, *mrc1Δ*::*Nat*, *iod^−^)* strain of the *japonicus* variety. Fortunately, these two varieties were compatible in meiotic intercrosses (Figure 3). We recently reported a >99.9% DNA sequence identity of the *mat2* and *mat3* genes of *versatilis* (Yu *et al.* 2013) with those of the *japonicus* variety (Rhind *et al.* 2011). Thus, it is not surprising to discover that the *versatilis* and *japonicus* varieties are genetically fertile when crossed with each other.

Analysis of this cross demonstrated that the *Nat^r^* drug resistance marker segregated by the expected four drug-resistant: four drug-sensitive patterns in each octad (Figure 3). Moreover, we were able to score this marker in just 12 hr of growth of replicates on the nourseothricin antibiotic-containing medium (Figure 3). However, scoring *mat1* alleles by replica-plating segregants to plates containing lawns of our mating-type tester strains, SJk2 and SjK3, with the aforementioned iodine-staining procedure, was only partially successful. This is a simple replica-plating procedure commonly used to score the *mat1* marker of *S. pombe* crosses (Bresch *et al.* 1968). In our cross between the *versatilis* and *japonicus* varieties, some segregants showed iodine-positive responses while others did not. Therefore, we determined the *mat1* marker of segregants by mating them to our mating-type testers, Sjk2 and Sjk3, and microscopically observing their ability to form zygotes. The expected 4 *mat1-P*: 4 *mat1-M* segregation pattern was found in all 12 octads we analyzed (Figure 3).

### The iodine-nonstaining phenotype of japonicus variety changed to the staining phenotype by crossing with the versatilis variety

Interestingly, the mating mix of the cross did not exhibit an iodine-positive staining phenotype despite containing plenty of ascospores. Therefore we conclude that the iodine-negative staining genotype (designated *iod*^-^) of *japonicus* variety is dominant to the iodine-positive genotype (*iod^+^*) of *versatilis* variety. However, from the cross we were able to choose segregants that showed iodine-positive reactions; in subsequent crosses with *versatilis* strains, their iodine-positive feature was propagated through mitosis and meiosis. For example, Sjk17 (a segregant of the cross discussed above; *h^+^*, *mat-2017*, *mrc1Δ*::*Nat*, *iod^+^*) and Sjk3 cross produced 38 Parental ditype: 7 Nonparental ditype: 39 Tetratype asci for segregation of the *mat1* and *mrc1* pair of genetic markers, a result indicating loose genetic linkage between them. Notably, the *mat1* marker in this cross was recorded by the replica-plating and iodine-staining procedure employed for research with *S. pombe*.

### Nuclei orientation is indeterminate in the *S. japonicus* meiosis

Interestingly, the two spores at each end of the ascus are “sisters” in the linear four-spored asci of the *S. pombe* fission yeast (Leupold 1950), whereas they are nonsisters in both *Saccharomycodes ludwigii* (Winge and Laustsen 1939; Yamazaki *et al.* 1976) and *Sc. cerevisiae* (Hawthorne 1955) budding yeasts. This difference results from two different types of nuclei distribution in a species-specific manner after second nuclear division in meiosis within the ascus. The two spindles of second meiotic division are arranged in a line in *S. pombe* but they lie parallel to each other in the long axis of ascogenous cells of *S. ludwigii* and *S. cerevisiae*. A minority of yeast asci has spores arranged in a linear array, called linear asci. The nuclei distribution can be determined genetically by analyzing segregation pattern of centromere-linked markers of spore segregants extracted from the linear asci. We likewise defined the nuclear location behavior during *S. japonicus* meiosis, although this yeast, in comparison to other yeasts, produces 8-spored asci due to a subsequent mitotic division of each of the four meiotic nuclei. Because *S. japonicus* cells mate at the cellular pole, nearly 20% of *S. japonicus* zygotes produce discernible linear asci (see one example of it in Figure 3). We dissected linear asci from a cross of Sjk10 (*mat1-P*, *mat2*/3*Δ*, *ade6*) with Sjk19 (*mat1-M*, *mat2*/3*Δ*) strains. This cross was designed to segregate four unlinked markers, of which, *ade6* (on chromosome 2) and *mat1* (on chromosome 1) markers are tightly linked to their respective centromeres (Furuya and Niki 2009; Rhind *et al.* 2011; Yu *et al.* 2013). The asci were carefully dissected and the spores were collected and analyzed in the order they had been originally arranged in each ascus. To analyze the data, we compared the genetic composition of four pairs of segregants derived from each ascus such that each pair represented adjoining spores in the ascus. Among the nine linear asci analyzed, 21 among 36 pairs were discordant for alleles of at least one among the four markers; two of them were centromere linked (Table 2). On the basis of these data we conclude that members of a pair do not always derive from sister nuclei and that the nuclei orientation during meiosis in the elongated ascogenous cell is indeterminate. Thus, *S. japonicus* arranges nuclear products in meiosis in an unordered fashion, a pattern very different from those of the three unrelated yeast species quoted above.

**Table 2:** Marker segregation in the linear asci of Sjk10 × Sjk19 cross

Ascus	Spore	*mat1*	*ade6*	*ura4*	*mrc1*	Ascus	Spore	*mat1*	*ade6*	*ura4*	*mrc1*	Ascus	Spore	*mat1*	*ade6*	*ura4*	*mrc1*
1	A	M	–	–	–	2	A	P	+	+	+	3	A	M	–	+	–
	B	M	–	–	–		B	P	+	–	–		**B**	M	–	+	–
	C	P	+	+	–		C	P	+	+	+		C	M	–	–	–
	D	M	–	+	+		D	P	+	–	–		D	P	+	–	+
	E	P	+	+	–		**E**	M	–	+	–		E	M	–	–	–
	F	M	–	+	+		F	M	–	–	+		F	P	+	–	+
	G	P	+	–	+		G	M	–	–	+		G	P	+	+	+
	H	P	+	–	+		H	M	–	+	–		H	P	+	+	+
4	A	M	+	+	–	5	A	M	+	+	+	6	A	P	–	–	+
	B	M	+	–	+		B	P	–	+	+		B	P	–	–	+
	C	M	+	+	–		**C**	M	+	+	+		C	P	–	–	+
	D	P	–	–	+		D	M	+	–	–		D	P	–	–	+
	E	M	+	–	+		E	P	–	+	+		E	M	+	+	–
	F	P	–	–	+		F	P	–	–	–		F	M	+	+	–
	G	P	–	+	–		G	M	+	–	–		G	M	+	+	–
	H	P	–	+	–		H	P	–	–	–		H	M	+	+	–
7	A	M	+	–	+	8	A	P	–	+	–	9	A	P	–	+	–
	B	M	+	–	+		B	M	+	–	+		B	P	–	+	–
	C	M	+	–	+		**C**	P	–	+	+		C	P	–	+	+
	D	M	+	–	+		D	M	+	–	–		D	P	–	+	+
	E	P	–	+	–		E	P	–	+	+		E	M	+	–	+
	F	P	–	+	–		F	M	+	–	–		F	M	+	–	–
	G	P	–	+	–		G	M	+	–	+		G	M	+	–	+
	H	P	–	+	–		H	P	–	+	–		H	M	+	–	–

Data of nine asci are displayed. The letters A to H indicate spore segregants of an ascus. Spores shown in bold letters were dead. The dead spore’s genotype was deduced from a segregant that did not have a matching sister spore genotype because each meiotic product undergoes mitosis to produce sister spores with identical genotype.

### The variety versatilis grows very fast

**Figure 4:**
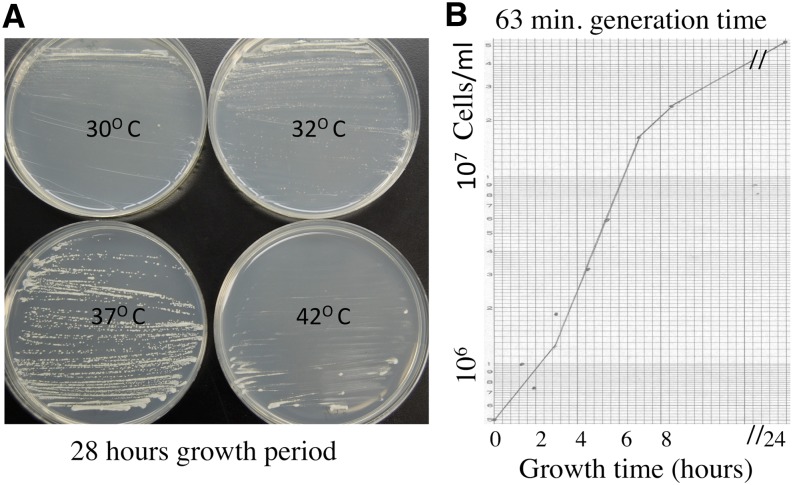
Growth of the Sjk2 strain. (A) The effect of temperature on the growth of Sjk2 colonies. The single-cell colony isolation procedure was performed on YEA medium-containing plates incubated at the indicated temperatures. The picture of plates was taken after 28 hr of growth. (B) The growth curve. The culture was grown in 50 mL of YEA broth medium contained in a 250-mL flask. The flask was shaken at 37° for aeration. The generation time calculated from the exponential phase of growth curve was 63 min.

*S. Japonicus* colonies grew best at 37° among indicated temperatures, which we tested (Figure 4A). Individual cells grew into colonies visible to the naked eye after growth for 28 hr. Supporting this fast colony-growth phenotype, cultures grew in liquid growth medium with a generation time of only 63 min (Figure 4B) compared with between 1.5 and 2.0 hr required by *S. pombe* and *S. cerevisiae*. For comparison, we derived generation time of 93 min from the previously published growth curve of variety *japonicus* (Rhind *et al.* 2011). Thus, *versatilis* cultures grow significantly faster than those of *japonicus* variety.

The reason for the iodine-negative phenotype of the *japonicus* variety remains to be determined in future studies. In our cross of the *versalitis* and *japonicus* varieties presented above, 12.3% segregants showed iodine-positive response. This result is consistent with the idea that *japonicus* variety’s *iod^−^* phenotype is due to at least two unlinked genes difference between the species. Nonetheless, as demonstrated here, the *vesatilis* and *japonicus* varieties can be intercrossed and strains with iodine-positive genetic constitutions can be easily derived through genetic crosses. Because of the feasibility of intercrossing these two varieties, recently developed research tools consisting of DNA-mediated transformation (Aoki *et al.* 2010), gene deletions and drug markers inserted at 500 Kb apart in the entire genome of the *japonicus* variety (Furuya *et al.* 2012) could be used for research with the *S. japonicus* organism. The material generated for research with the *japonicus* variety should be combined by genetic crosses with the highly useful iodine-positive trait of the *versatilis* variety for future research with the *S. japonicus* organism. Already, the *japonicus* variety has been exploited as an organism to define the mechanism of dimorphic transition from yeast to hyphal growth, which is often associated with pathogenicity in some fungi (Furuya and Niki 2012); *S. japonicus* is not a human pathogen (Furuya and Niki 2009).

Because there is only 55% identity at the amino acid level between 1:1 orthologs of *S. japonicus* proteins (an organism with the 44% GC content) with those of *S. pombe* (an organism with the 36% GC content), these organisms have diverged far apart in evolution (Rhind *et al.* 2011). Indicating evolutionary divergence, we found that the *S. pombe* cells do not mate with *S. japonicus* cells. Both *S. pombe* and *Sc. cerevisiae* produce 4-spored asci and go through mitosis without nuclear breakdown. In comparison, *S. japonicus* produces 8-spored asci (Figure 2), and it goes through mitosis with a partial nuclear membrane breakdown (Robinow and Hyams 1989). Investigations with evolutionarily distinct organisms have been the key to discovering conserved mechanisms of biological processes. Both *S. pombe* and *Sc. cerevisiae* grow with a generation time between 1.5 and 2.0 hr and accomplishing their genetic cross requires a period of more than a week. In comparison with those popular research organisms, *S. japonicus* var. *versatilis* grew with a generation time of only 63 min (Figure 4B) and the meiotic analysis of a genetic cross was completed in just 2.5 days time (Figures 2 and 3). This author has more than 40 years of experience working on the biology of both *Sc. cerevisiae* (Klar 2010) and *S. pombe* (Klar 2007). On the basis of previous experience working with other yeasts and because of features described here that are deemed very much conducive for research, one can predict that *S. japonicus* species would become a popular experimental organism in the near future.
